# Supplementary material for: Impaired memory B-cell recall responses in the elderly following recurrent influenza vaccination
Source: PLoS One. 2021 Aug 5;16(8):e0254421. doi: 10.1371/journal.pone.0254421 (PMC8341655; doi:10.1371/journal.pone.0254421)
Supplement: S1 Fig — A-F) Profile response to the H1N1 vaccine strain. G-L) Profile response to the H3N2 vaccine strains. High-HAI antibodies in Q1, high non-HAI in Q2, strong HAI-Abs in Q3 and non-responders in Q4. Young-adult participants are depicted as red dots and elderly in blue. Doted lines represent the cohort’s average for rHA-specific IgG pre-vaccination (horizontal) and the generally correlated 1:40 protective serum HAI titer (vertical). Changes in the proportion of participants in each quadrant over time were assess by a Chi-square test (χ2). (DOCX) [file pone.0254421.s001.docx]

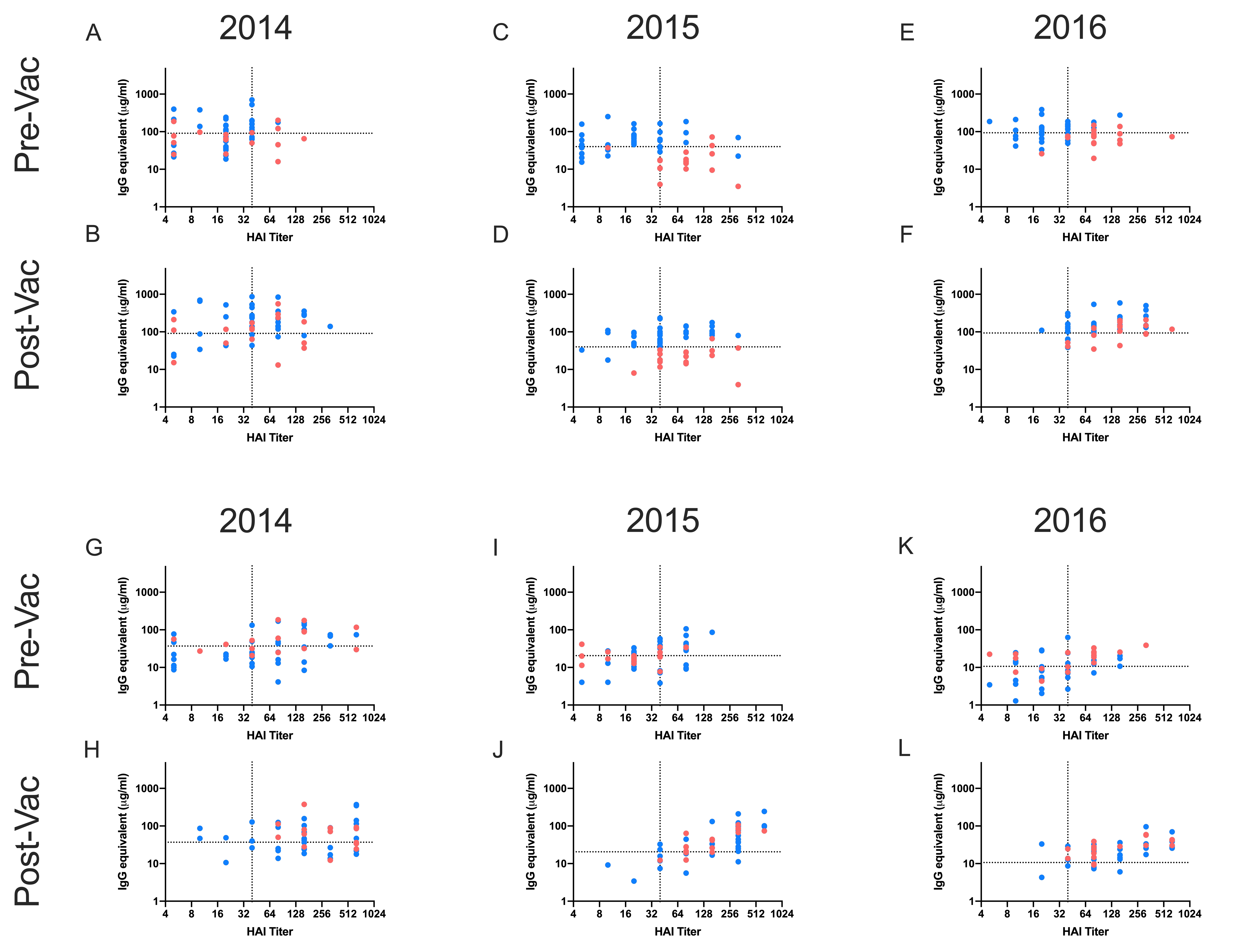


**S1 Fig:** Biparametric quadrant analysis of HAI titer and rHA-specific IgG (μg/mL) from 50 subjects (16 young-adult and 34 elderly) vaccinated for three consecutive years with standard of care inactivated influenza vaccine. A-F) Profile response to the H1N1 vaccine strain. G-L) Profile response to the H3N2 vaccine strains. High-HAI antibodies in Q1, high non-HAI in Q2, strong HAI-Abs in Q3 and non-responders in Q4. Young-adult participants are depicted as red dots and elderly in blue. Doted lines represent the cohort’s average for rHA-specific IgG pre-vaccination (horizontal) and the generally correlated 1:40 protective serum HAI titer (vertical). Changes in the proportion of participants in each quadrant over time were assess by a Chi-square test (χ^2^).
