# Supplementary material for: Impaired memory B-cell recall responses in the elderly following recurrent influenza vaccination
Source: PLoS One. 2021 Aug 5;16(8):e0254421. doi: 10.1371/journal.pone.0254421 (PMC8341655; doi:10.1371/journal.pone.0254421)
Supplement: S2 Fig — Changes in frequency of plasmablasts of total B-cells 7 and 21–28 days after vaccination in young-adult (A) and elderly (B) participants. (DOCX) [file pone.0254421.s002.docx]

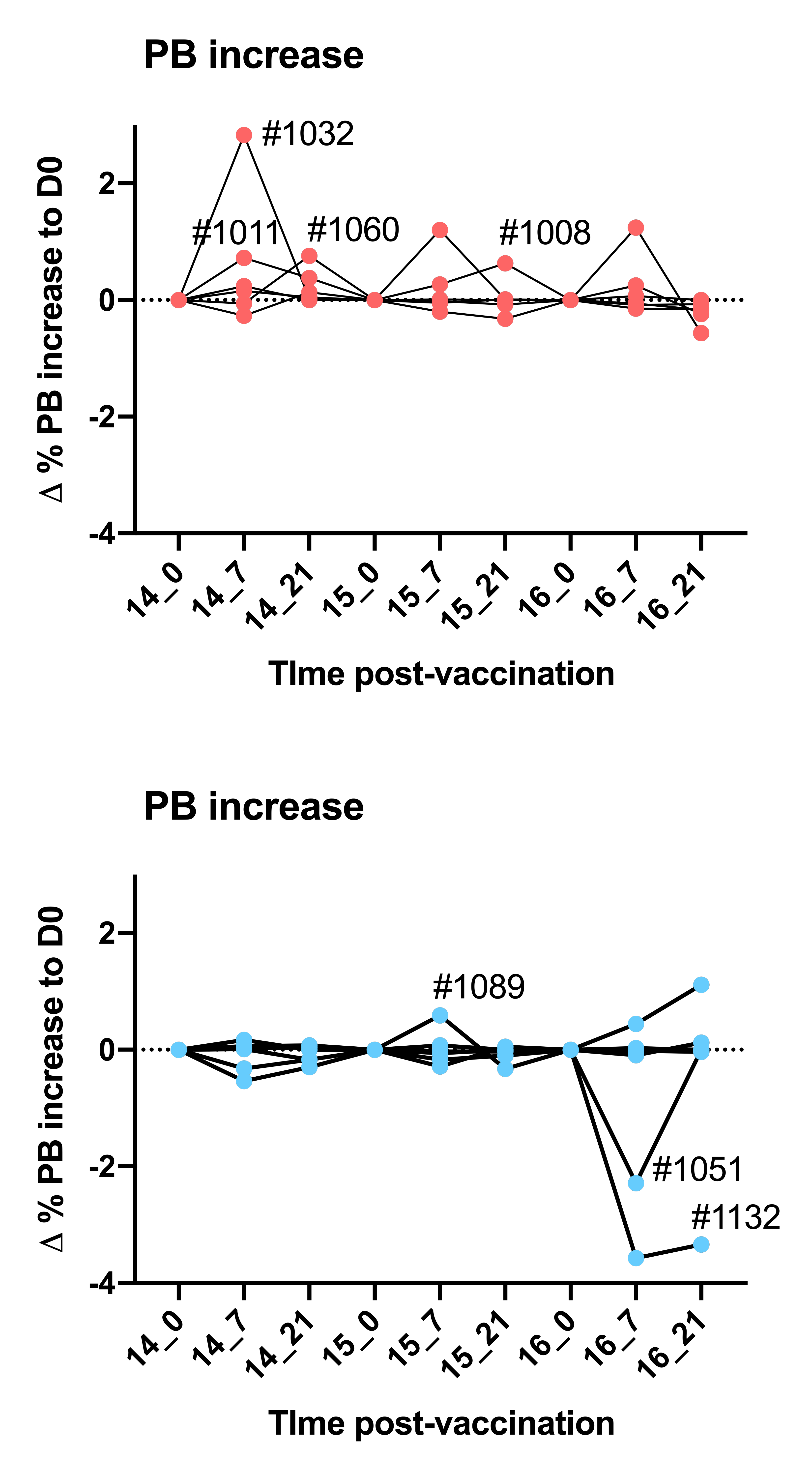


**S2 Fig:** Changes in frequency of plasmablasts of total B-cells 7 and 21-28 days after vaccination in young-adult (A) and elderly (B) participants.
