# Supplementary material for: Impaired memory B-cell recall responses in the elderly following recurrent influenza vaccination
Source: PLoS One. 2021 Aug 5;16(8):e0254421. doi: 10.1371/journal.pone.0254421 (PMC8341655; doi:10.1371/journal.pone.0254421)
Supplement: S3 Fig — (DOCX) [file pone.0254421.s003.docx]

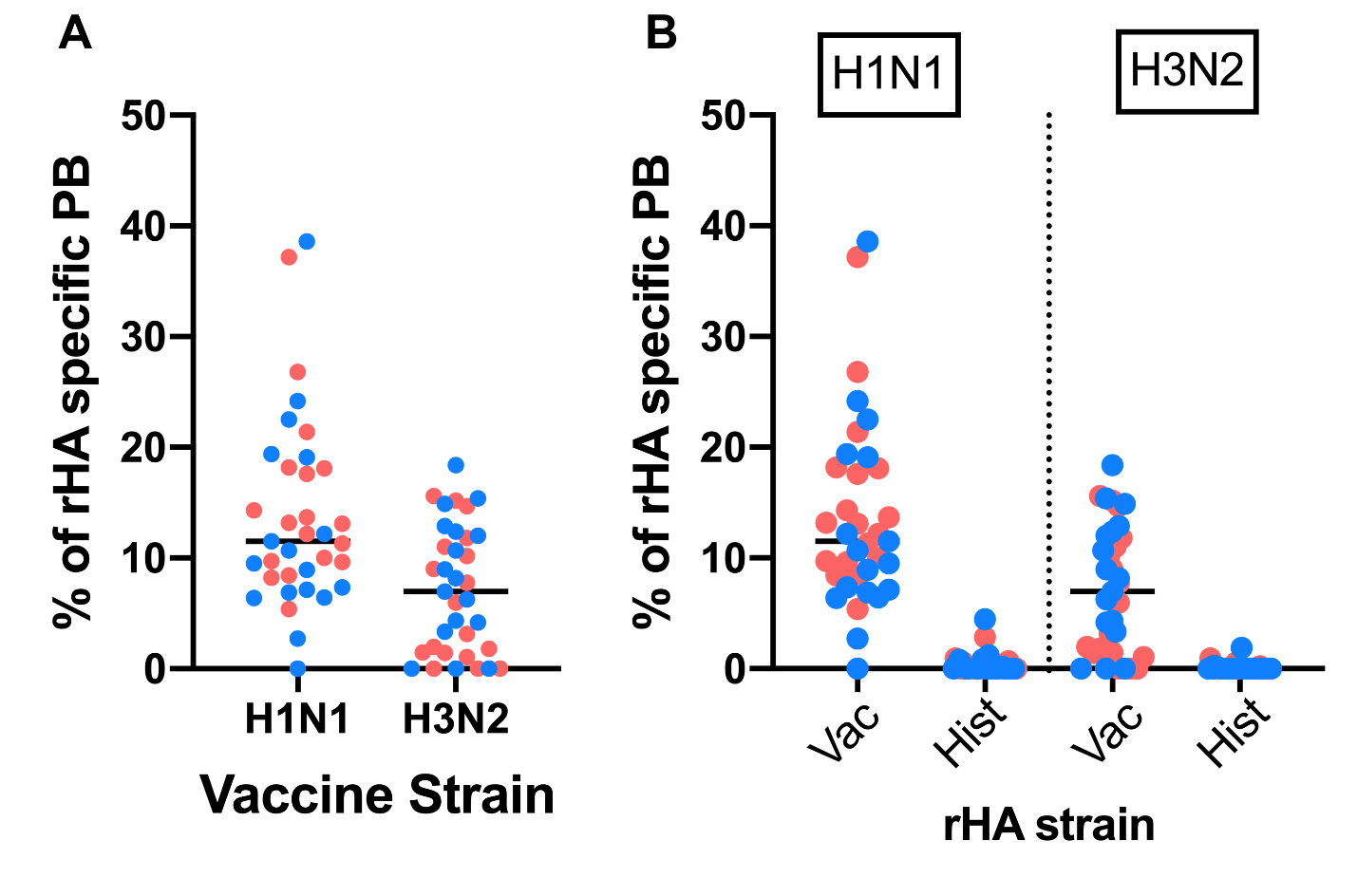


**S3 Fig:** Frequency of rHA-reactive plasmablasts against H1N1 and H3N2 vaccine strains in young (red) and elderly (blue) subjects vaccinated over three consecutive years.
