# Supplementary material for: Impaired memory B-cell recall responses in the elderly following recurrent influenza vaccination
Source: PLoS One. 2021 Aug 5;16(8):e0254421. doi: 10.1371/journal.pone.0254421 (PMC8341655; doi:10.1371/journal.pone.0254421)
Supplement: S4 Fig — A-C) Serological IgG antibodies against rHA from current H1N1 vaccine strain and 4 historical seasonal H1N1 virus strains (1983–2007) in three young subjects vaccinated for three consecutive years. Colors represent antigenic similarities between H1 rHA. D-F) Serological IgG antibody levels against rHA from the current H3N2 vaccine strains and 5 historical seasonal H3N2 virus strains (1999–2011) in three young subjects vaccinated for three consecutive years. Colors represent antigenic similarities between H3 rHA. (DOCX) [file pone.0254421.s004.docx]

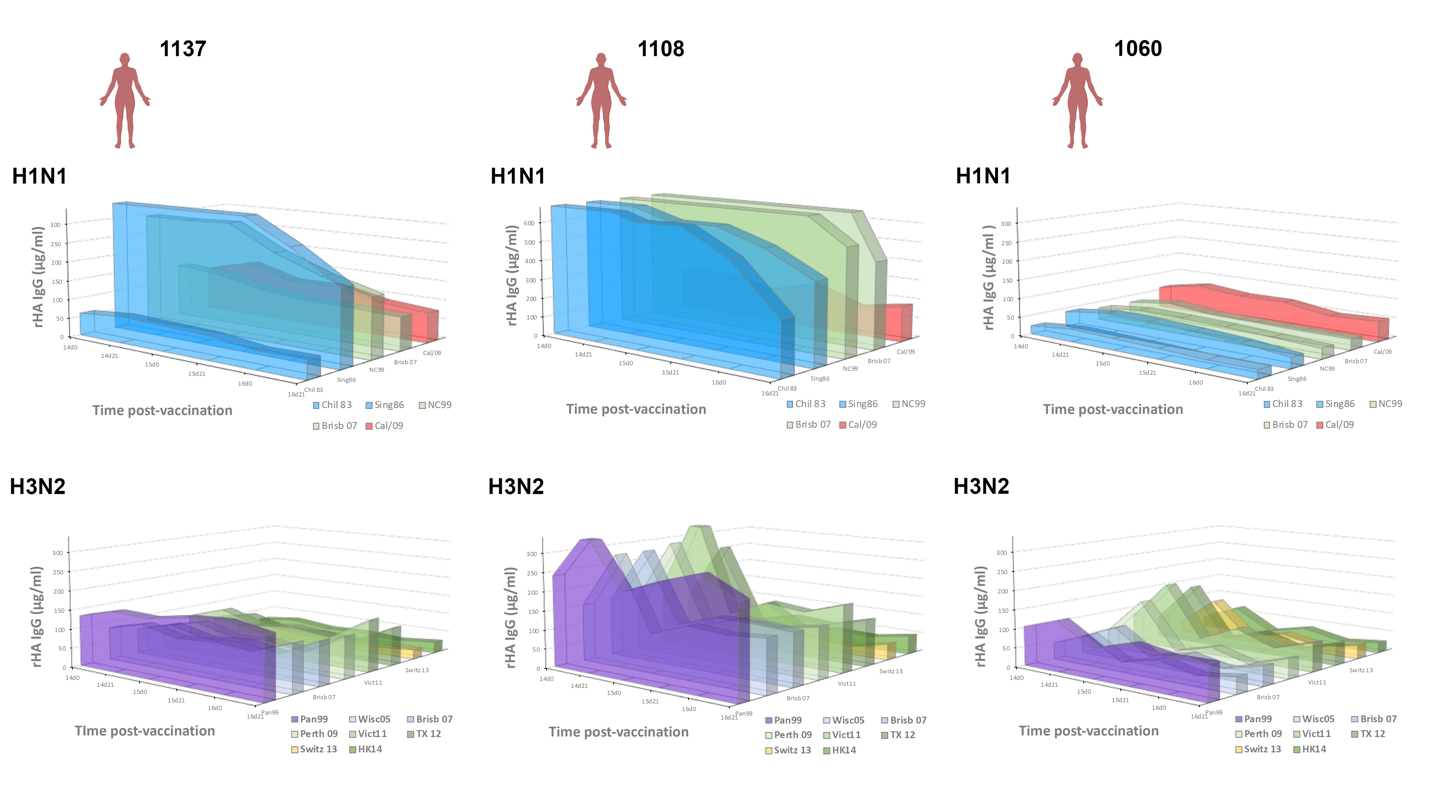


**S4 Fig:** Serological antibody landscape in young-adult participants vaccinated for three consecutive years. A-C) Serological IgG antibodies against rHA from current H1N1 vaccine strain and 4 historical seasonal H1N1 virus strains (1983-2007) in three young subjects vaccinated for three consecutive years. Colors represent antigenic similarities between H1 rHA. D-F) Serological IgG antibody levels against rHA from the current H3N2 vaccine strains and 5 historical seasonal H3N2 virus strains (1999-2011) in three young subjects vaccinated for three consecutive years. Colors represent antigenic similarities between H3 rHA.
