# Supplementary material for: Impaired memory B-cell recall responses in the elderly following recurrent influenza vaccination
Source: PLoS One. 2021 Aug 5;16(8):e0254421. doi: 10.1371/journal.pone.0254421 (PMC8341655; doi:10.1371/journal.pone.0254421)
Supplement: S5 Fig — (DOCX) [file pone.0254421.s005.docx]

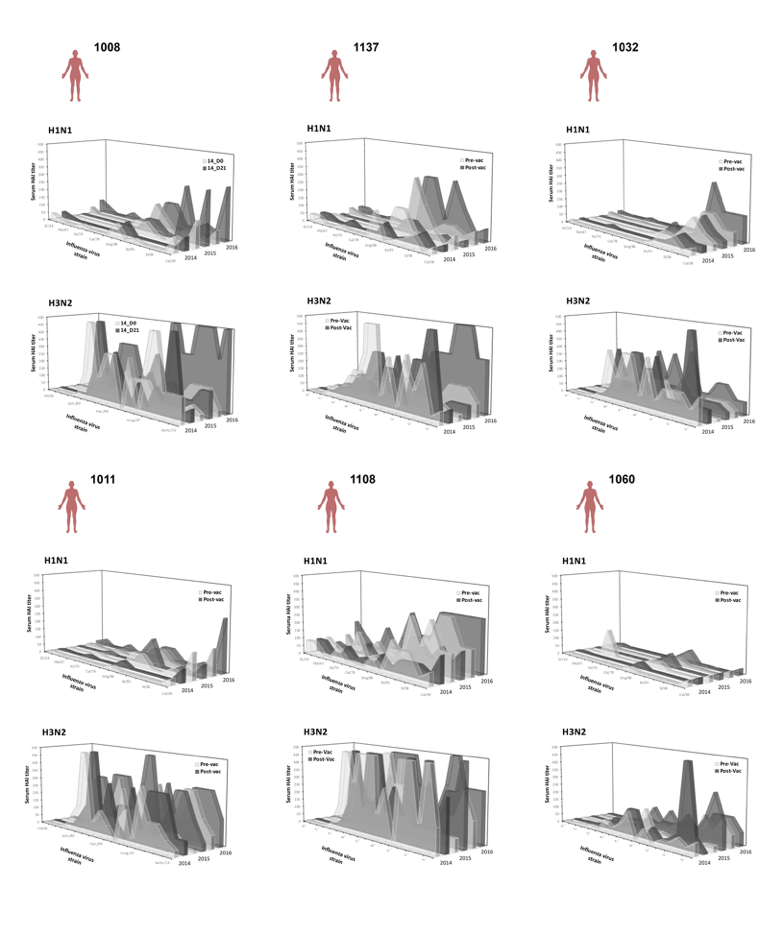


**S5 Fig:** HAI antibody landscape against a broad panel of H1N1 (A-C and G-I) or H3N2 (D-F and J-L) in 6 young-adult participants vaccinated for three consecutive years.
