# Supplementary material for: Impaired memory B-cell recall responses in the elderly following recurrent influenza vaccination
Source: PLoS One. 2021 Aug 5;16(8):e0254421. doi: 10.1371/journal.pone.0254421 (PMC8341655; doi:10.1371/journal.pone.0254421)
Supplement: S8 Fig — (DOCX) [file pone.0254421.s008.docx]

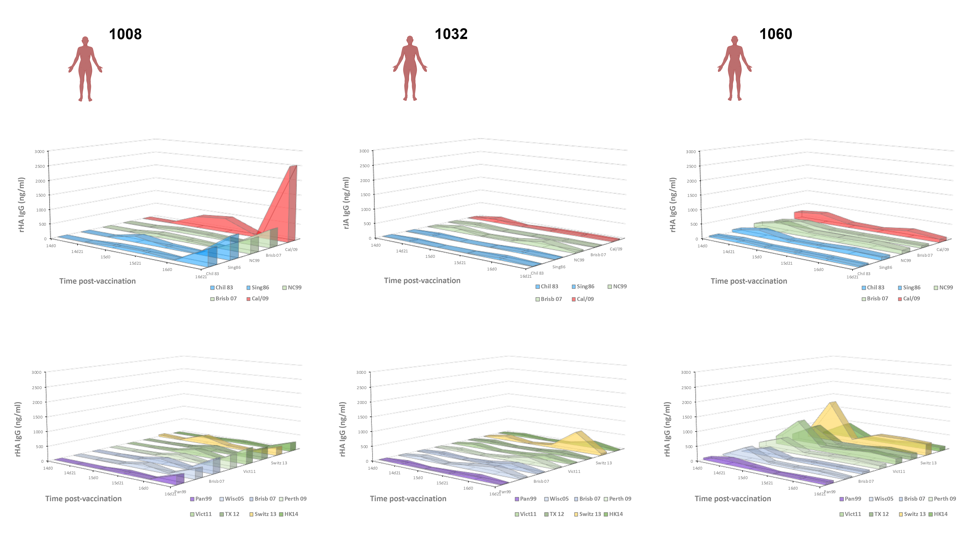


**S8 Fig:** Bmem-derived IgG antibodies against rHA from current vaccine and historical seasonal influenza virus strains in three young adult participants vaccinated for three consecutive years.
