# Supplementary material for: Impaired memory B-cell recall responses in the elderly following recurrent influenza vaccination
Source: PLoS One. 2021 Aug 5;16(8):e0254421. doi: 10.1371/journal.pone.0254421 (PMC8341655; doi:10.1371/journal.pone.0254421)
Supplement: S10 Fig — Resulting transformed data was used for panels G-L in Figs 4 and 5. (DOCX) [file pone.0254421.s010.docx]

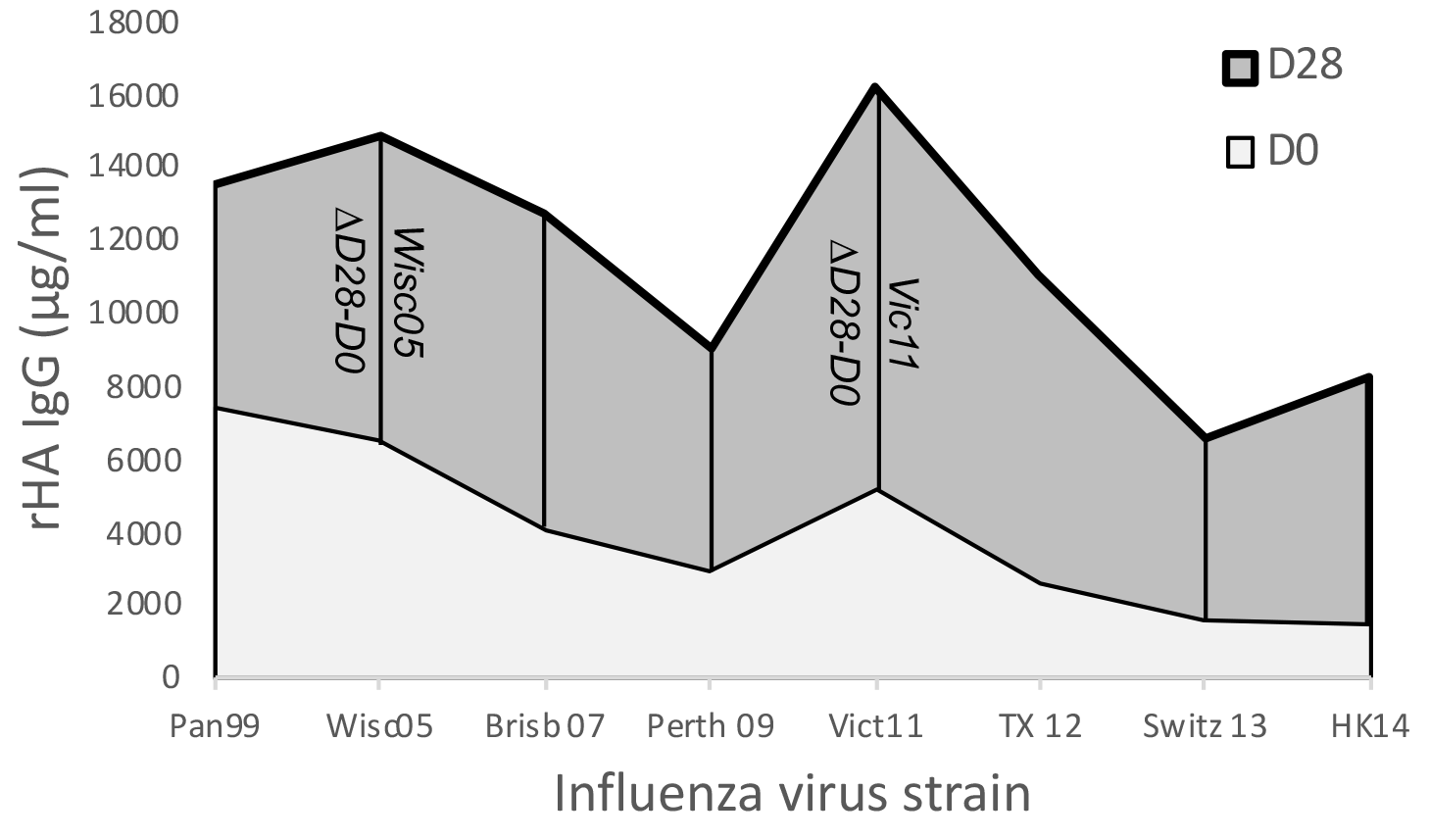


**S10 Fig:** Illustrative approach to calculate vaccine induced rHA-reactive antibodies every year in each analyzed participant (D#1132 in 2014 shown). Resulting transformed data was used for panels G-L in figure 4 and 5.
