# Supplementary material for: Impaired memory B-cell recall responses in the elderly following recurrent influenza vaccination
Source: PLoS One. 2021 Aug 5;16(8):e0254421. doi: 10.1371/journal.pone.0254421 (PMC8341655; doi:10.1371/journal.pone.0254421)
Supplement: S1 Table — (DOCX) [file pone.0254421.s011.docx]

**S1 Table:** Key resources and reagents

| **REAGENT or RESOURCE** | **SOURCE** | **IDENTIFIER** |
| --- | --- | --- |
| **Antibodies** | | |
| IgD BV421 | BioLegend | Cat# 348226 |
| CD20 BV650 | BioLegend | Cat# 302334 |
| CD3 FITC | BioLegend | Cat# 300306 |
| CD14 FITC | BioLegend | Cat# 301804 |
| CD38 PE-Dazzel | BioLegend | Cat# 303538 |
| CD27 PE-Cy7 | BioLegend | Cat# 124216 |
| CD19 APC-Fire | BioLegend | Cat# 302258 |
|  |  |  |
| **Bacterial and Virus Strains** | | |
| IAV-H1N1 (A/South Carolina/1918) | Laboratory of Ted Ross, University of Georgia | Lot BCL-112915SC1918VLP |
| IAV-H1N1 (A/Weiss/1/1943) | VIRAPUR | Lot VP-H1517B |
| IAV-H1N1 (A/Fort Monmouth/1/1947) | VIRAPUR | Lot VP-I1528A |
| IAV-H1N1 (A/Denver/1/1957) | VIRAPUR | Lot VP-H1517A |
| IAV-H1N1 (A/New Jersey/8/1976) | VIRAPUR | Lot VP-G1509B |
| IAV-H1N1 PR8 (A/USSR/90/1977) | VIRAPUR | Lot VP-G1509C |
| IAV-H1N1 PR8 (A/Brazil/11/1978) | VIRAPUR | Lot VP-G1501A |
| IAV-H1N1 PR8 (A/Chile/1/1983) | VIRAPUR | Lot VP-J1509A |
| IAV-H1N1 (A/Singapore/6/1986) | VIRAPUR | Lot VP-L1509C |
| IAV-H1N1 (A/Texas/39/1991) | VIRAPUR | Lot VP-I1530C |
| IAV-H1N1 PR8 (A/Beijing/262/1995) | VIRAPUR | Lot VP-H1517C |
| IAV-H1N1 PR8 (A/New Caledonia/20/1999) | VIRAPUR | Lot VP-G1509A |
| IAV-H1N1 PR8 (A/Solomon Islands/3/2006) | VIRAPUR | Lot VP-F1522A |
| IAV-H1N1 PR8 (A/Brisbane/59/2007) | VIRAPUR | Lot VP-F1524A |
| IAV-H1N1 PR8 (A/California/07/2009) | VIRAPUR | Lot VP-F1512B |
| IAV-H1N1 (A/Michigan/45/2015) | VIRAPUR | Lot VP-K1610A |
| IAV-H3N2 (A/Hong Kong/8/1968) | VIRAPUR | Lot VP-L1503A |
| IAV-H3N2 (A/Port Chalmers/1/1973) | VIRAPUR | Lot VP-K1516D |
| IAV-H3N2 PR8 (A/Texas/1/1977) | Laboratory of Ted Ross, University of Georgia | Lot AB-2315TX77MDCK1 |
| IAV-H3N2 PR8 (A/Mississippi/1/1985) | Laboratory of Ted Ross, University of Georgia | Lot BCL-12315M85MDCK1 |
| IAV-H3N2 (A/Sichuan/60/1989) | Laboratory of Ted Ross, University of Georgia | BCL-012615SCH89EP1 |
| IAV-H3N2 (A/Shangdong/9/1993) | Laboratory of Ted Ross, University of Georgia | Lot BCL-2615S93MDCK1 |
| IAV-H3N2 PR8 (A/Nanchang/933/1995) | VIRAPUR | Lot VP-K1622A |
| IAV-H3N2 (A/Sydney/5/1997) | Laboratory of Ted Ross, University of Georgia | Lot BCL-020116SY97EP1 |
| IAV-H3N2 (A/Panama/2007/1999) | VIRAPUR | Lot VP-L1503B |
| IAV-H3N2 (A/New York/55/2004) | VIRAPUR | Lot VP-C1729E |
| IAV-H3N2 (A/Wisconsin/67/2005) | VIRAPUR | Lot VP-J1519C |
| IAV-H3N2 PR8 (A/Uruguay/716/2007) | VIRAPUR | Lot VP-I1606C |
| IAV-H3N2 (A/Perth/16/2009) | VIRAPUR | Lot VP-H1507A |
| IAV-H3N2 (A/Victoria/361/2011) | VIRAPUR | Lot VP-J1519B |
| IAV-H3N2 (A/Texas/50/2012) | VIRAPUR | Lot VP-I1508B |
| IAV-H3N2 (A/Switzerland/9715293/2013) | VIRAPUR | Lot VP-J1506B |
| IAV-H3N2 PR8 (A/Hong Kong/4801/2014) | VIRAPUR | Lot VP-E1603A |
|  |  |  |
| **Biological Samples** | | |
| Human PBMCs (D0, D7, D21-28) | This paper | Reviewed and approved by the IRB of the University of Georgia |
| Serum samples (D0, D21-28) | Nuñez *et al.* 2017 (23) |  |
|  |  |  |
|  |  |  |
| **Chemicals, Peptides, and Recombinant Proteins** | | |
| H1N1 A/Chile/1/1983 (Chile/83) rHA | Laboratory of Ted Ross, University of Georgia | Sautto et al., 2018, Sautto et al., 2020 |
| H1N1 A/Singapore/6/1986 (Sing/86) rHA | Laboratory of Ted Ross, University of Georgia | Sautto et al., 2018, Sautto et al., 2020 |
| H1N1 A/New Caledonia/20/1999 (NC/99) rHA | Laboratory of Ted Ross, University of Georgia | Sautto et al., 2018, Sautto et al., 2020 |
| H1N1 A/Brisbane/59/2007 (Brisb/07) rHA | Laboratory of Ted Ross, University of Georgia | Sautto et al., 2018, Sautto et al., 2020 |
| H1N1 A/California/07/2009 (CA/09) rHA | Laboratory of Ted Ross, University of Georgia | Abreu et al., 2020 |
| H1N1 A/Michigan/45/2015 (MI/15) rHA | Laboratory of Ted Ross, University of Georgia | Sautto et al., 2018, Sautto et al., 2020 |
| H1N1 A/Brisbane/02/2018 (Brisb/18) rHA | Laboratory of Ted Ross, University of Georgia | N/A |
| H3N2 A/Panama/07/1999 (Pan/99) rHA | Laboratory of Ted Ross, University of Georgia | N/A |
| H3N2 A/Wisconsin/67/2005 (WI/05) rHA | Laboratory of Ted Ross, University of Georgia | N/A |
| H3N2 A/Brisbane/10/2007 (Brisb/07) rHA | Laboratory of Ted Ross, University of Georgia | N/A |
| H3N2 A/Perth/16/2009 (Perth/09) rHA | Laboratory of Ted Ross, University of Georgia | N/A |
| H3N2 A/Victoria/361/2011 (Vic/11) rHA | Laboratory of Ted Ross, University of Georgia | N/A |
| H3N2 A/Texas/50/2012 (TX/12) rHA | Laboratory of Ted Ross, University of Georgia | N/A |
| H3N2 A/Switzerland/9715293/2013 (Switz/13) rHA | Laboratory of Ted Ross, University of Georgia | N/A |
| H3N2 A/Hong Kong/4801/2014 (HK/14) rHA | Laboratory of Ted Ross, University of Georgia | N/A |
