# Supplementary material for: Impaired memory B-cell recall responses in the elderly following recurrent influenza vaccination
Source: PLoS One. 2021 Aug 5;16(8):e0254421. doi: 10.1371/journal.pone.0254421 (PMC8341655; doi:10.1371/journal.pone.0254421)
Supplement: Attachment — Submitted filename: Response Letter.Abreu PLOS ONE.2nd Revision.docx [file pone.0254421.s013.docx]

June 21, 2021

Anna Mentsl

PLOS ONE

Dear Editor

To address the reviewer’s comment, we added a small study limitations paragraph at the end of the discussion (highlighted in yellow) clearly stating the impact of small sample size on the overall data interpretation.

This should be sufficient to alert the reader for the lack statistical differences between groups in some data.

Best regards,

Ted M. Ross, Ph.D.

GRA Eminent Scholar in Infectious Diseases

Director - Center for Vaccines and Immunology

Professor - Department of Infectious Diseases

University of Georgia
